# Robust mitotic entry is ensured by a latching switch

**DOI:** 10.1242/bio.20135199

**Published:** 2013-07-26

**Authors:** Chloe Tuck, Tongli Zhang, Tamara Potapova, Marcos Malumbres, Béla Novák

**Affiliations:** 1 Oxford Centre for Integrative Systems Biology, Department of Biochemistry, South Parks Road, Oxford OX1 3QU, UK; 2 Stowers Institute for Medical Research, Kansas City, MO 64110, USA; 3 Cell Division and Cancer Group, Spanish National Cancer Research Centre (CNIO), Melchor Fernández Almagro 3, E-28029 Madrid, Spain

**Keywords:** G2/M transition, Mitotic entry, Mitotic collapse, Greatwall kinase, Spatial control of mitosis, Cell cycle

## Abstract

Cell cycle events are driven by Cyclin dependent kinases (CDKs) and by their counter-acting phosphatases. Activation of the Cdk1:Cyclin B complex during mitotic entry is controlled by the Wee1/Myt1 inhibitory kinases and by Cdc25 activatory phosphatase, which are themselves regulated by Cdk1:Cyclin B within two positive circuits. Impairing these two feedbacks with chemical inhibitors induces a transient entry into M phase referred to as mitotic collapse. The pathology of mitotic collapse reveals that the positive circuits play a significant role in maintaining the M phase state. To better understand the function of these feedback loops during G2/M transition, we propose a simple model for mitotic entry in mammalian cells including spatial control over Greatwall kinase phosphorylation. After parameter calibration, the model is able to recapture the complex and non-intuitive molecular dynamics reported by Potapova et al. (Potapova et al., 2011). Moreover, it predicts the temporal patterns of other mitotic regulators which have not yet been experimentally tested and suggests a general design principle of cell cycle control: latching switches buffer the cellular stresses which accompany cell cycle processes to ensure that the transitions are smooth and robust.

## Introduction

The eukaryotic cell cycle is the process during which a mother cell duplicates all its components and divides them more or less evenly between two daughter cells, providing them with the necessary information and machinery to repeat the process. In order to keep the genetic content constant over subsequent generations, chromosomes are alternately replicated in S phase and then equally segregated between two daughter cells in M phase. Between these two cell cycle events are two gap phases (G1 and G2) before and after S, respectively. Both G1 and G2 allow the cell to assess the readiness to proceed to the next cell cycle phase (Morgan, 2006).

Cell cycle events are controlled by phosphorylation through Cyclin dependent kinases (CDKs; Cdk1, Cdk 2, Cdk 4 and Cdk6) and dephosphorylation catalysed by counter-acting phosphatases (e.g. PP2A-B55). CDKs require activatory subunits called Cyclins (Cyclin A, Cyclin B, Cyclin D, Cyclin E etc.), most of whose levels oscillate during the cycle. In humans, Cyclin B is the major activatory subunit required by Cdk1 to promote mitosis and most of Cyclin B (CycB) is synthesised over G2 (Brizuela et al., 1989). However, during S and G2 phase the Cdk1 subunit of the complex is phosphorylated (Draetta et al., 1988), which inhibits Cdk1:CycB activity and stops premature entry into mitosis (Krek and Nigg, 1991). Inhibitory phosphorylations are carried out by two kinases, Wee1 and Myt1. Wee1 preferentially phosphorylates and inhibits Cdk1 at Tyrosine 15 (McGowan and Russell, 1993; Parker et al., 1995; Watanabe et al., 1995). However Wee1 is phosphorylated by Cdk1 during mitosis, which inhibits its kinase activity (Parker et al., 1995; Watanabe et al., 1995). Myt1 preferentially phosphorylates and inhibits Cdk1 on Threonine 14 and is also inhibited by Cdk1 phosphorylation in mitosis (Booher et al., 1997). Cdk1:CycB also phosphorylates and activates an activatory phosphatase, called Cdc25, during mitotic progression. Cdc25 removes the inhibitory phosphorylations on Cdk1 (Hoffmann et al., 1993) and its depletion can cause G2 arrest in human cells (Galaktionov and Beach, 1991). Recent experiments suggest that M phase entry requires concomitant inhibition of a Cdk1 counter-acting phosphatase (PP2A-B55), which is achieved by Greatwall-kinase dependent activation of phosphatase inhibitors (Gharbi-Ayachi et al., 2010; Mochida et al., 2010).

The feedbacks between Cdk1:CycB and the enzymes regulating Cdk1 inhibitory phosphorylations (Wee1/Myt1 and Cdc25) control the activation of Cdk1:CycB. These feedbacks create switch-like activation and inactivation thresholds for Cdk1:CycB. The activation and inactivation thresholds of Cdk1:CycB for mitotic entry and exit are not the same, a phenomena called hysteresis (Novak and Tyson, 1993). The M phase state can be maintained with a lower Cdk1 activity than the activation threshold, therefore M phase is a self-maintained state. Hence, mitotic entry is an irreversible process controlled by a bistable switch (Pomerening et al., 2003; Sha et al., 2003). Removal of the bistable switch in human cells causes rapid cycles between a mitotic like state without proper exit and cytokinesis (Pomerening et al., 2008).

In a recent paper, the functional significance of these positive feedbacks was directly tested in HeLa Cells. When both Wee1/Myt1 and Cdc25 were inhibited in early G2 phase, cells prematurely entered into mitosis. However, cells were unable to maintain their mitotic state after nuclear envelope breakdown (NEBD) and the mitotic phenotype was reverted. Compromising Cdk1's activatory positive feedback loops by inhibition of Wee1/Myt1 and Cdc25 makes mitotic entry reversible, and this phenomenon has been coined ‘mitotic collapse’ (Potapova et al., 2011).

Mitotic collapse clearly indicates that cells experience some challenges during mitotic entry. Normally, the challenges are handled by the positive feedbacks through Cdc25 and Myt1/Wee1. However if the feedbacks are weakened, the cellular challenges could result in serious problems. The phenomenon of mitotic collapse raises three related questions. What could be the cellular challenges during mitotic entry? How do cells normally handle these challenges and why do cells fail to do so during mitotic collapse?

To answer these questions, we present a dynamical model for the G2/M transition of mammalian cells. To handle the complexity brought about by NEBD, the model considers spatial control of Greatwall phosphorylation, which allows us to recapitulate the intriguing dynamics reported by Potapova et al. in quantitative details (Potapova et al., 2011). We propose that NEBD might cause a cellular stress during mitotic entry, which would usually be buffered by the bistability in Cdk1 activation. When the bistability is compromised (such as by loss of positive feedbacks through Wee1/Myt1 and Cdc25), the cellular buffering capacity is impaired and mitotic entry becomes reversible and mitotic collapse takes place. This case-study reinforces the claim that bistability is an essential property for irreversible cell cycle transitions (Novak et al., 2007).

### Model construction

#### The wiring diagram

**Fig. 1.**
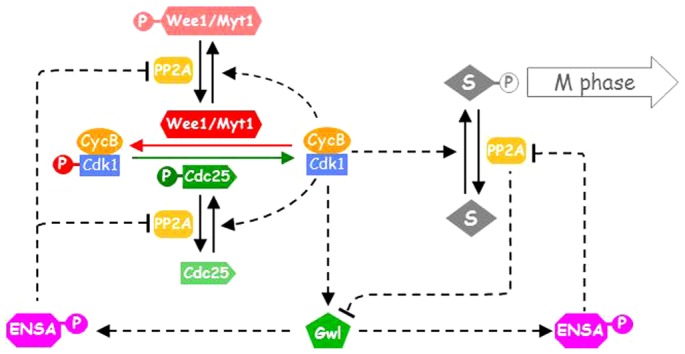
The wiring diagram of the mitotic control model. Wee1/Myt1 and Cdk1:CycB inhibit each other, while Cdc25 and Cdk1:CycB activate each other. Cdk1:CycB phosphorylates Greatwall kinase (Gwl). The phosphatase PP2A-B55 is assumed to dephosphorylate Wee1/Myt1, Cdc25 and Gwl. The active Gwl-P activates ENSA and thus represses PP2A-B55. When ENSA is dephosphorylated, it no longer binds to PP2A-B55. A generic mitotic substrate (S), phosphorylated and dephosphorylated by Cdk1:CycB and PP2A-B55, respectively, is an indicator of the mitotic progression.

The molecular mechanism of our model used to analyse the kinetics of mitotic entry of HeLa cells in the experiments of Potapova et al. (Potapova et al., 2011) is summarized in a wiring diagram (Fig. 1). In the experiments, nocodazole was used to disrupt the mitotic spindle which activates the mitotic checkpoint that blocks CycB degradation by Anaphase Promoting Complex/Cyclosome (APC/C). To simplify the model, we assume that CycB is only synthesized in S/G2 phases and its level becomes constant once cells enter into mitosis. Since Cdk1 is in excess of CycB (Arooz et al., 2000), the formation of Cdk1:CycB complex is limited by CycB availability, therefore we only monitor CycB levels which will correspond to the Cdk1:CycB complex. Myt1/Wee1 kinases and Cdk1:CycB phosphorylate and inhibit each other in a double-negative feedback (Booher et al., 1997; Parker et al., 1995; Watanabe et al., 1995). Meanwhile, Cdc25 and Cdk1:CycB activate each other in a positive feedback loop (Hoffmann et al., 1993).

Cdk1:CycB also phosphorylates and activates Greatwall kinase (Blake-Hodek et al., 2012; Yu et al., 2006), which is abbreviated as Gwl in this model. Greatwall-kinase phosphorylates and activates two phosphatase inhibitors, Endosulphine and Arpp19 (Gharbi-Ayachi et al., 2010; Kim et al., 2012; Mochida et al., 2010). These two inhibitors are referred here simply as ENSA. The phosphorylated forms of these inhibitors (ENSA-P) bind and inhibit PP2A-B55, which dephosphorylates Cdk1:CycB target proteins, including Wee1/Myt1 and Cdc25 (Clarke et al., 1993; Mochida et al., 2009).

Since Greatwall is a Cdk1:CycB substrate, it has been proposed that it is dephosphorylated by PP2A-B55 (Domingo-Sananes et al., 2011). Though this assumption has not been directly confirmed by experiments yet, it is supported by the following experimental evidence. If protein synthesis is repressed and Cdc25 is depleted in an interphase *Xenopus* extract, Cdk1 activity is low and Gwl is unphosphorylated. Despite of the low Cdk1 activity, Gwl becomes abruptly phosphorylated when PP2A-B55 is repressed by addition of phosphorylated Endosulfine or Arpp19 (see figure 2B of Gharbi-Ayachi et al. (Gharbi-Ayachi et al., 2010)). This experiment implies that PP2A-B55 dephosphorylates Gwl. Therefore Gwl and PP2A-B55 inhibit each other which adds another positive feedback loop to the network.

#### Spatial controls and the effect of nuclear envelope breakdown

The majority of Wee1 is located in the nucleus (Heald et al., 1993) but Myt1 is located in the cytoplasm (Liu et al., 1997). In this regard, an inhibitory kinase is available to phosphorylate Cdk1 in both the nucleus and cytoplasm. Hence, in the current model we use Wee1/Myt1 to represent the overall Cdk1 inhibitory kinase activity. Similarly, Cdc25C is available in cytoplasm (Dalal et al., 1999) while Cdc25A is in the nucleus (Källström et al., 2005), so we use Cdc25 to represent their cumulative phosphatase activity.

Cdk1:CycB is activated and imported into the nucleus just before nuclear envelope breakdown (Gavet and Pines, 2010; Santos et al., 2012). However, significant Cdk1:CycB remains in the cytoplasm, which could be important to ensure synchronisation of cytoplasmic and nuclear mitotic events (Gavet and Pines, 2010). We assume that active Cdk1:CycB shuttles between the cytoplasm and the nucleus, being enriched in the nucleus until NEBD takes place.

Most Greatwall kinase resides in the nucleus during interphase when Cdk1:CycB activity is low (Burgess et al., 2010; Voets and Wolthuis, 2010) suggesting that unphosphorylated Greatwall mainly accumulates in the nucleus. Since active Cdk1:CycB enters the nucleus before NEBD, nuclear phosphorylation of Gwl becomes enhanced by co-localization of substrate (Gwl) and enzyme (Cdk1:CycB). Assuming that Gwl phosphorylation is localized to the nucleus before NEBD, but dephosphorylation is not, the change in phosphorylated Gwl concentration ([Gwlp]) over time is given by:
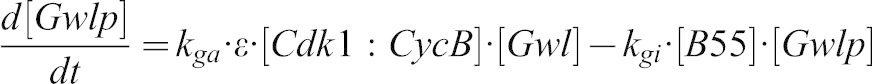
where *k_ga_* and *k_gi_* are the rate constants for Gwl activation (phosphorylation) and inactivation (dephosphorylation) respectively. The ‘ε’ factor describes the fold increase in rate of Gwl phosphorylation as a consequence of colocalization of Cdk1:CycB and unphosphorylated Gwl in the nucleus. The value of ‘ε’ is proportional to the inverse of the nucleo-cytoplasmic ratio. The nucleo-cytoplasmic ratio is around 10% in eukaryotic cells (Huber and Gerace, 2007; Joerger and Fersht, 2007), which gives a value for ‘ε’ around 10 in interphase. In principle, at NEBD the value of ‘ε’ should be reduced to one if nuclear Cdk1:CycB and Gwl get quickly distributed throughout the whole cell. However, neither of them occupies the entire cell volume during mitosis, but rather they preferentially accumulate around chromosomes and the spindle (Burgess et al., 2010; Voets and Wolthuis, 2010). To reflect this inhomogeneous sub-cellular localization, we reduce ε to 25% upon NEBD. To simplify the presentation we scale ‘ε’ to one in interphase and reduce it to 0.25 in mitosis after NEBD.

ENSA is evenly distributed in the nucleus and in the cytoplasm, at least in Drosophila (Drummond-Barbosa and Spradling, 2004). Because PP2A-B55 substrates localize both in the cytoplasm and in the nucleus, we assume that both ENSA and PP2A-B55 are evenly distributed throughout the cell.

We also supplement the model with a downstream mitotic substrate (S) that is phosphorylated and dephosphorylated by Cdk1 and PP2A-B55, respectively. The phosphorylation state of this substrate determines whether the cell is in G2 phase (unphosphorylated) or in mitosis (phosphorylated). We use the phosphorylation state of this substrate (substrate-P or S-P) as a proxy for nucleolin phosphorylation that is measured in the experiments of Potapova et al. (Potapova et al., 2011). We also assume in the model that NEBD takes place when the phosphorylation state of S exceeds 70%.

All the above assumptions are made to simplify the model without compromising its main purpose: to understand the dynamical processes of normal mitotic entry and mitotic collapse.

## Results

### Temporal dynamics during normal mitotic entry and mitotic collapse

The dynamics of the molecular regulatory system controlling mitotic entry in HeLa cells was recently characterized by Potapova et al. after synchronization in S phase with double thymidine block (Potapova et al., 2011). Most cells completed DNA replication around six hours after thymidine wash-out. Further mitotic progression of these G2 cells was monitored by means of histone-H3 phosphorylation in the presence of a spindle poison (nocodazole). In order to investigate the role of Cdk1 inhibitory phosphorylation, the Wee1/Myt1 inhibitor (PD0166285) and the Cdc25 inhibitor (NSC663284) or their combination was applied six hours after thymidine release, i.e. at the beginning of G2 phase. Most G2 cells entered into mitosis within 10 hours after thymidine release in the absence of any chemical inhibitor. The Cdc25 inhibitor blocked mitotic entry during this time window, which is indicated by the lack of histone-H3 phosphorylation during the next four hours after S phase completion. On the other hand, Wee1/Myt1 inhibition accelerated histone-H3 phosphorylation, and mitotic entry were seen within one hour after S phase completion. Adding the two (Wee1/Myt1 and Cdc25) inhibitors together also advanced histone-H3 phosphorylation, but only transiently because the mitotic state collapsed.

**Fig. 2.**
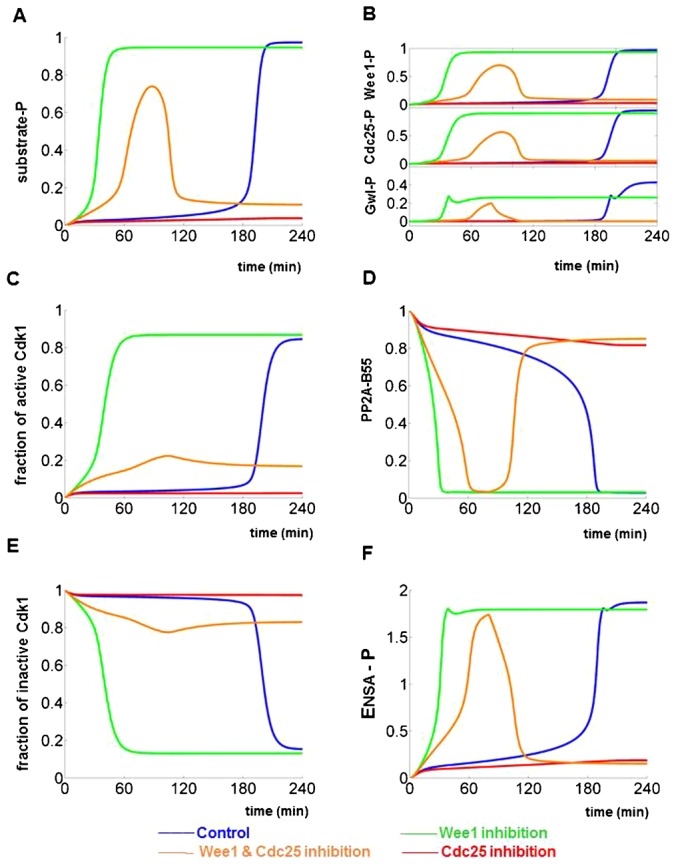
Time series simulations of mitotic progression and mitotic collapse. The time series simulations start from early G2 state with low CycB levels, low Cdk1:CycB activity and high PP2A-B55 activity. The temporal patterns of mitotic regulators are plotted in four different situations: normal mitotic progression (blue), Wee1/Myt1 (green), Cdc25 (red) and Wee1/Myt1 and Cdc25 double (orange) inhibition. Phosphorylated mitotic substrate (**A**), phosphorylated Wee1, Cdc25 and Gwl (**B**), fraction of active Cdk1 (**C**), active PP2A-B55 (**D**), fraction of inactive (phosphorylated) Cdk1:CycB (**E**) and active (phosphorylated) Ensa-P (**F**). The model simulations are plotted in this way to facilitate the direct comparison to the following experimental figures in Potapova et al.: figure 5A,C (A), figure 5B (B), figure 6A (C) and figure 5C (E) (Potapova et al., 2011). Model simulations in panels D and F have not been confirmed experimentally yet, therefore they are predictions.

We have used our model to simulate the time-course experiments in figure 5 of Potapova et al. (Potapova et al., 2011). Numerical simulations were started with initial conditions characteristic for cells in early G2 phase (Wee1/Myt1 and PP2A-B55 active, Cdk1:CycB, Cdc25 and Gwl inactive, and low level of CycB etc.; supplementary material Table S1). The initial CycB level (0.5 a.u.) was not enough to activate Cdk1 in control cells. Cdk1:CycB activation (measured by H1 kinase activity in the experiments) and mitotic substrate phosphorylation required three hours of CycB synthesis during G2 phase (Fig. 2A,C). Cdc25 inhibition prevented phosphorylation of mitotic substrate and Cdk1 activation despite increasing CycB levels. Inhibition of Wee1/Myt1 with or without the Cdc25 inhibitor causes premature substrate phosphorylation and mitotic entry. Since Wee1/Myt1 inhibited cells can enter into M phase with the CycB level present initially (0.5), we simply simulated their mitotic progression with constant levels of CycB, which is consistent with the experimental data. Mitotic substrate phosphorylation (S-P) stays high when Wee1/Myt1 is inhibited which corresponds to a stable mitotic state (Fig. 2A). In contrast, mitotic substrate phosphorylation is transient in case of the double inhibition (Fig. 2A), consistent with the mitotic collapse in the experiments. The model simulations have also recaptured the phosphorylation patterns of Wee1, Cdc25 and Gwl (compare figure 5B of Potapova et al. (Potapova et al., 2011) and simulations in Fig. 2B). Remember that Wee1/Myt1, Cdc25 and Gwl are more than just passive readouts of the Cdk1/PP2A-B55 activity ratio, because they are also active regulators of the Cdk1:CycB kinase and the PP2A-B55 phosphatase.

During mitotic collapse, the transient phosphorylation of Cdk1:CycB substrates results from transient elevation of the Cdk1/PP2A-B55 ratio. This transient increase of the kinase/phosphatase ratio is mainly caused by the change in PP2A-B55 rather than Cdk1 activity (Fig. 2C,D). The simulated Cdk1 activity (Fig. 2C) confirms the experiments in figure 6A of Potapova et al. (Potapova et al., 2011), while the change of phosphatase activity (Fig. 2D) is a prediction of our model that waits for experimental verification.

To provide a comprehensive picture, the current model is used to simulate the temporal pattern of inhibitory phosphorylated Cdk1:CycB (pre-MPF, Fig. 2E) and that of phosphorylated ENSA (ENSA-P, Fig. 2F). The simulation of pre-MPF agrees well with the experimental finding in figure 5C of Potapova et al. (Potapova et al., 2011), while the predicted Ensa dynamics awaits further experimental testing.

In summary, our kinetic model reproduces the temporal dynamics that have been carefully observed during normal mitotic entry and mitotic collapse. Besides reproducing the temporal patterns of experimentally measured mitotic regulators, the model also predicts dynamic changes of components that have not been followed. However, the numerical simulations alone are insufficient to provide a satisfactory mechanism of mitotic collapse. To complement the time series simulations and to provide a deeper mechanistic insight, we analyse the model with the mathematical tool of bifurcation diagrams.

### Explanation of mitotic collapse by dynamical systems theory

**Fig. 3.**
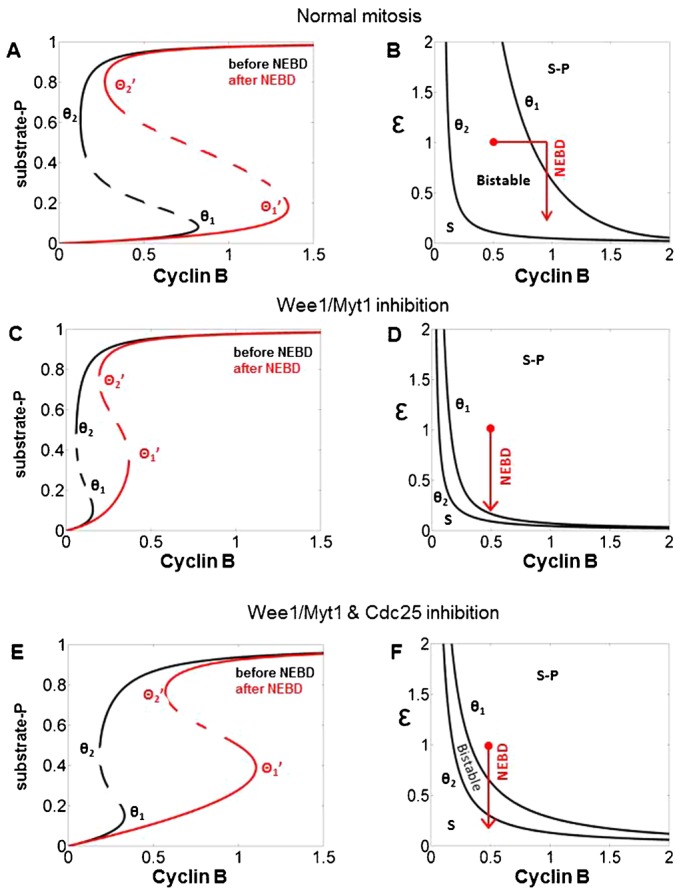
Bifurcation analysis of mitotic progression and mitotic collapse. One- and two-parameter bifurcation diagrams are shown for normal mitotic progression (**A**,**B**), Wee1/Myt1 inhibition (**C**,**D**) and Wee1/Myt1 and Cdc25 double inhibition (**E**,**F**). One parameter bifurcation diagrams (A,C,E). CycB level is used as the bifurcation parameter while phosphorylated mitotic substrate indicates the state of the mitotic controls system. The black and the red curves represent the bifurcation diagrams before (ε = 1) and after (ε = 0.25) nuclear envelope breakdown, respectively. Solid lines represent stable while dashed lines unstable steady states. Normal mitotic progression (A), Wee1/Myt1 inhibition (C) and Wee1/Myt1 and Cdc25 double inhibition (E). The cyclin thresholds (labelled by Greek θ) for mitotic substrate phosphorylation and dephosphorylation are indicated with subscripts one and two, respectively. Between the two corresponding cyclin thresholds the mitotic susbtrate has two alternative stable steady states (G2 and M). Two parameter bifurcation diagrams (B,D,F). CycB and epsilon (ε) are chosen as the two parameters. The left black curve records the values of the cyclin thresholds (θ_2_ values) for the mitotic substrate (S) dephosphorylation at different ε values. Its value is θ_2_ when ε = 1 and its value is θ_2_′ when ε = 0.25. The right black curve records the values of the cyclin thresholds for the mitotic susbtrate phosphorylation, which is θ_1_ when ε = 1 and θ_1_′ when ε = 0.25. The bistability region is between these two curves. The red lines record the time dependent trajectory of the system during mitotic progression. During normal mitotic progression (B) the trajectory starts from the G2 state in the bistable regime (red dot) and moves to the right at constant epsilon (ε) value until it crosses the cyclin threshold for phosphorylation of the mitotic substrate. As cells enter mitosis, NEBD triggers epsilon to decrease so the trajectory drops vertically and ends up the M phase state of the bistable regime. After Wee1/Myt1 inactivation (D), mitotic entry is triggered without the requirement of CycB production and the only stable steady state is in M phase (red dot). NEBD causes ε to decrease which drives the system vertically down, but it does not leave the M phase regime. When the two inhibitors for Wee1/Myt1 and Cdc25 are added after S phase (F), the system trajectory also drops vertically. The decrease of epsilon (ε) causes the system to enter the region with unphosphorylated mitotic substrate (mitotic collapse).

It is informative to analyse the model for mitotic entry using one-parameter bifurcation diagrams. A one-parameter bifurcation diagram plots stable and unstable attractors (e.g. steady states) of a dynamical system as functions of an arbitrary ‘parameter’. We choose CycB as a bifurcation ‘parameter’; although it is not constant during the cell cycle, it is the slowest dynamic variable of the mitotic control network. The influence of CycB (bifurcation parameter) on the dynamical network can be illustrated by any of the molecular regulators. We choose arbitrarily the generic mitotic substrate (S), which corresponds to phosphorylated nucleolin and histone-H3 in the experiments, to characterize the state of the system (Fig. 3A). At low CycB levels, the mitotic substrate is unphosphorylated therefore this steady state refers to the G2 phase of the cell cycle. At high CycB levels, Cdk1 is active and mitotic substrate is fully phosphorylated as in M phase (Fig. 3A, black curve). The positive circuits of the regulatory network define a bistable switch with two different CycB thresholds for mitotic entry (θ_1_) and mitotic exit (θ_2_). Between these two cyclin thresholds the control system can occupy either of the two stable states (G2 or M), and it will depend on the history of the system as to which one it settles on. For that reason, the bistable switch is also called as a hysteresis switch. The CycB threshold for mitotic entry is sensitively dependent on the level of Greatwall-kinase; therefore, our model is consistent with the experimental data showing that Gwl reduces the Cdk1 activity required for NEBD (Hara et al., 2012).

The black curve on Fig. 3A characterizes the system with unphosphorylated Gwl concentrated in the nucleus. Once the cell reaches the θ_1_ cyclin threshold for the G2/M transition, it starts its vertical movement to the upper steady state corresponding to M phase. During mitotic entry the nuclear envelope breaks down, which slows down Gwl phosphorylation because of dispersion of Cdk1:CycB and Gwl and weakens PP2A-B55 inhibition within the cell. The drop in PP2A-B55 inhibition makes activation of Cdk1 more difficult, which is manifested in the increase of both cyclin thresholds (θ_1_′ and θ_2_′ of the red curve). In the model, we use the decrease of a single parameter (ε) to represent the effects of NEBD. Therefore the cellular response to NEBD is better illustrated on a two-parameter bifurcation diagram where we plot the two cyclin thresholds (the left and the right edges of the S-shaped curve) over a range of ε values (Fig. 3B).

On this diagram, the left curve connects the points of mitotic exit thresholds (θ_2_) at different values of ε. Therefore, the area left and below this curve represents G2 phase of the cycle where mitotic substrate (S) is unphosphorylated. The right curve corresponds to the cyclin thresholds for mitotic entry (θ_1_) and the area above and to the right of this curve represents M phase where the mitotic substrate is phosphorylated (S-P). Between the two curves the system is bistable with low (G2) and high (M) mitotic substrate phosphorylation states coexisting. This should be imagined as a pleated sheet from a top view where the folds of the sheet correspond to the curves on the diagram.

Early G2 phase cells (CycB = 0.5 and ε = 1) start their mitotic journey from the low steady state in the bistable region because they have sub-threshold CycB levels. They move to the right by CycB synthesis (red horizontal line) and they enter mitosis once they pass the θ_1_ curve. Since mitotic entry is accompanied by NEBD, which weakens nuclear phosphorylation of Gwl, ε gets decreased (vertical red line). The system falls back into the bistable region, but the mitotic substrate remains phosphorylated (S-P) because the θ_2_ threshold is not crossed and the mitotic state persists in the bistable regime.

When Wee1/Myt1 kinases are inhibited both CycB thresholds (θ_1_ and θ_2_) are decreased and the bistable region also becomes greatly reduced (Fig. 3C). Since the cyclin threshold for mitotic entry (θ_1_) is lower, cells can now enter into mitosis at a CycB level (CycB = 0.5) which was not permissive in the absence of the inhibitor. Once the phosphorylation of mitotic substrate reaches a threshold (0.7 in the model), NEBD takes place. Now both cyclin thresholds are increased due to the drop in ε, however this increase is relatively slight (Fig. 3C, red curve). Since the cyclin threshold for mitotic entry (θ_1_) stays below the actual CycB level, cells keep on progressing towards highly phosphorylated mitotic substrate state and settle there. On the two parameter bifurcation diagram the trajectory becomes a vertical line (Fig. 3D), with both initial and final states in the highly phosphorylated substrate state (S-P), which is M phase of the cell cycle. In summary, inactivation of inhibitory kinases advances mitotic entry at low CycB level, and mitotic entry is still irreversible.

When Wee1/Myt1 and Cdc25 are both inhibited, cyclin thresholds are decreased as well. Since the hysteresis effect relies on the positive circuits controlling Cdk1 activation, the bistable region also becomes greatly reduced (Fig. 3E). Similar to the Wee1/Myt1 inhibition case, cells can now enter into mitosis at a lower level of CycB (0.5). At NEBD both cyclin thresholds are increased (Fig. 3E, red curve), but in this case the cyclin threshold for mitotic exit (θ_2_′) becomes larger than the threshold for mitotic entry (θ_1_). The gap created between the two thresholds (θ_1_ and θ_2_′) destabilizes the mitotic state and reverts the cell back to G2 phase with unphosphorylated mitotic subtrates (S), which corresponds to mitotic collapse.

Again, the cellular fate is better revealed by plotting the time dependent trajectory of the system on the ‘CycB–ε’ two-parameter bifurcation diagram (Fig. 3F, red line). The same initial conditions as control cells (CycB = 0.5 and ε = 1) define a point in the M phase regime. Activation of Cdk1 and phosphorylation of mitotic substrates accompany M phase entry and cause NEBD. NEBD reduces the value of ε which pushes the system down through the small region of bistability into the G2 state with mitotic substrates (S) dephosphorylated. The journey from the phosphorylated mitotic substrates region into the unphosphorylated one corresponds to phenomena of mitotic collapse.

### Suppression of mitotic collapse

**Fig. 4.**
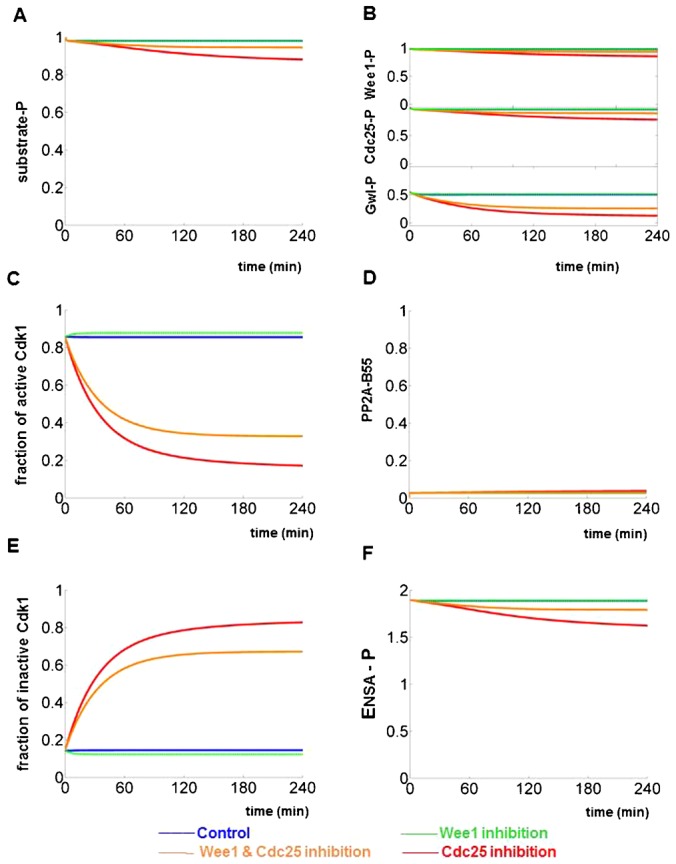
Simulating the responses of mitotic cells to chemical inhibitors. (**A–F**)The model is first allowed to reach a mitotic state, with high CycB level (1.3) and low PP2A-B55 activity, and time series simulations are started from this steady state. As in Fig. 2, different panels record the dynamics of different molecules and four colours are used to represent four different experimental conditions. The dynamics of phosphorylated mitotic substrate (A) is directly comparable to figure 6B of Potapova et al. (Potapova et al., 2011). All the other panels are model predictions.

The bifurcation diagram on Fig. 3E provides a clear indication that mitotic collapse only happens in a restricted window of CycB levels. The CycB level must be larger than the threshold for mitotic entry (θ_1_ is at CycB = 0.35 on the black the curve of Fig. 3E) in order to bring about mitosis including NEBD in the first place. This requirement determines the lower boundary of the restricted CycB range. On the other hand, the CycB level must be smaller than the threshold for mitotic exit (θ_2_′ is at CycB = 0.55 at on the red curve of Fig. 3E) after NEBD. The lower and the upper limits of CycB levels suggest that mitotic collapse can take place after Wee1/Myt1 and Cdc25 double inhibition only in a short window of the cell cycle in early G2 phase. This conclusion is fully confirmed by experimental data in figure 6B of Potapova et al. (Potapova et al., 2011). When cells were first arrested in mitosis by nocodazole before treatment with Wee1/Myt1 and Cdc25 inhibitors, the mitotic state was stable for at least 4 hours. The time series simulations can nicely recapture the results of this experiment as well as predict the temporal dynamics of unseen mitotic regulators (Fig. 4). Surprisingly, the mitotic state is resistant against Cdc25 inhibition only as well (Fig. 4; figure 6B of Potapova et al. (Potapova et al., 2011)), because in M phase Wee1/Myt1 kinase are kept inactive by Cdk1:CycB. In summary, these results suggest that high CycB level suppresses mitotic collapse because the positive circuits of Cdk1:CycB activation are strong even if their strength is compromised by inhibitors.

Potapova et al. were also able to suppress mitotic collapse by inhibiting PP2A with okadaic acid (OA) (Potapova et al., 2011). Synchronized HeLa cells at the S/G2 boundary were treated with inhibitors of Wee1/Myt1 and Cdc25 followed by okadaic acid (OA) one hour later. Addition of the PP2A inhibitor (OA) was able to stabilize the mitotic state and thereby suppress mitotic collapse at least for the next 4 hours (figure 6C of Potapova et al. (Potapova et al., 2011)). This experiment strongly suggests that mitotic collapse is the consequence of misregulated ‘activation’ of PP2A during M phase.

The time series simulations with the model recapitulate the experimental results, as PP2A inhibition stabilises phosphorylation of mitotic substrates (supplementary material Fig. S1). In addition, the model predicts sustained phosphorylation of Wee1, Cdc25 and Gwl in the absence of PP2A-B55 activity (supplementary material Fig. S1). Despite the almost complete phosphorylation of Wee1 and Cdc25, the H1 kinase activity is predicted to be low due to the inhibition of Cdc25 by NSC663284 (supplementary material Fig. S1C). Partial Cdk1 dephosphorylation observed in figure 6C of Potapova et al. (Potapova et al., 2011) is also captured by our time series simulation (supplementary material Fig. S1D).

## Discussion

During progression through the cell cycle, cells undergo multiple transitions and qualitative changes to their physiological state. In general, there are five transitions during the eukaryotic cell cycle: Start (Restriction Point), G1/S, G2/M, meta/anaphase transitions and mitotic exit. It is important that all of these transitions are unidirectional and the cell does not revert back to the previous phase because that would jeopardise the strict alternation of chromosome replication and then segregation. In our view, all the eukaryotic cell cycle transitions are made unidirectional (irreversible) by underlying bistable switches (Novak et al., 2007; Verdugo et al., 2013). This proposal has been validated experimentally for all the cell cycle transitions of the budding yeast cell cycle (Charvin et al., 2010; Cross et al., 2002; Holt et al., 2008; López-Avilés et al., 2009) and for the G2/M transition in *Xenopus* oocytes (Pomerening et al., 2003; Sha et al., 2003). It is an important question whether the design principle of bistable cell cycle transitions is an evolutionally conserved property of the eukaryotic cell cycle control mechanisms. For this reason, we have studied the dynamics of the G2/M transition of mammalian cells which was carefully analysed experimentally by Potapova et al. (Potapova et al., 2011). Bistability of the G2/M transition could arise from the positive circuits regulating Cdk1:CycB activation through Wee1/Myt1 and Cdc25. Inhibition of both Wee1/Myt1 and Cdc25 early in G2 phase advanced cells into mitosis but progression though M phase was aborted. This has been named mitotic collapse (Potapova et al., 2011). Since inhibition of the enzymes responsible for inhibitory phosphorylations weakens bistability, this observation already supports the existence of a hysteresis switch. However it remains to be answered why cells exit prematurely from mitosis and what is the role of phosphatase regulation in this process.

According to our model, mitotic entry of mammalian cells is controlled by a bistable switch controlling Cdk1:CycB activation and inactivation of its counter-acting phosphatase, PP2A-B55. In G2 phase of the cell cycle, Cdk1 is kept inactive by inhibitory phosphorylation while PP2A-B55 is active. The accumulation of CycB pulls the switch when the threshold for mitotic entry is reached at the G2/M transition. Therefore the rise of CycB is a trigger pulling signal that turns on the switch by activating Cdk1 and inhibiting PP2A-B55. During the process of mitotic entry, nuclear envelope breakdown (NEBD) creates a cellular ‘stress’ that can jeopardise the G2/M transition. We propose that NEBD slows down the phosphorylation of Greatwall-kinase, which is critical for effective inhibition of PP2A-B55 and therefore of mitotic entry. During mitotic entry, this weakening of PP2A-B55 inhibition is normally compensated by the positive circuits driving Cdk1:CycB activation. If the positive circuits are compromised by Wee1/Myt1 and Cdc25 inhibitors, then PP2A-B55 overcomes Cdk1 activity and the mitotic state gets destabilized prematurely. We are not claiming that the slow-down of Gwl phosphorylation by NEBD is the only stress during mitotic progression. Another obvious ‘stress’ during mitotic progression is the degradation of Cyclin A by APC/C, since Cdk1:Cyclin A is helping Cdk1:CycB to be activated before mitosis. Cyclin A degradation is not controlled by the mitotic checkpoint and it takes place during prometaphase which implies that Cdk1:CycB alone must then maintain the high Cdk1 state for further mitotic progression. Nevertheless, we cannot conclude that mitotic collapse is caused by Cyclin A degradation, as its level does not appear to drop during mitotic collapse (figure 5C of Potapova et al. (Potapova et al., 2011)).

Interestingly enough, fission yeast cells with simultaneous inactivation of Wee1 and Cdc25 also undergo occasional mitotic collapses (Sveiczer et al., 1999). Cells of *wee1^ts^ cdc25Δ* double mutant show quantized cell cycles with random transitions among one, two and three units of cycle time (Sveiczer et al., 1996). The extended cycle times are the consequences of one or two unsuccessful attempts to execute mitosis after which cells reset back to early G2 phase and try to enter into M phase again later (Sveiczer et al., 1999). The probability of successful mitosis increases with cell size which presumably correlates with higher level of the Cdc13 mitotic cyclin.

We speculate that the requirement of bistability to suppress cellular stresses during cell cycle transition is not specific for the G2/M transition, but rather a generic phenomenon of the eukaryotic cell cycle control. Another example is the meta-to-anaphase transition, when cohesins holding sister-chromatids together are cleaved. Cohesins are important to provide tension against bipolar spindle forces during prometaphase and the lack of tension activates error correction and the mitotic checkpoint at this stage of mitosis. Yet cohesin cleavage does not reactivate error correction and the mitotic checkpoint during anaphase. This strongly suggests an underlying bistable switch (He et al., 2011). We propose that cellular ‘stresses’ accompanying eukaryotic cell cycle transitions may have been the selective forces necessary for underlying bistable switches to have evolved.

## Materials and Methods

### Model simulation and purpose driven parameter estimation

During the model construction, the molecular network in Fig. 1 was converted into a set of ordinary differential equations. To avoid unjustified nonlinearity, the model uses mass action kinetics only. The purpose of the current model is to understand the quantitative dynamics and the qualitative picture of mitotic entry and mitotic collapse. To achieve this goal, we adapt a trial and error method to estimate the model parameters. Starting from an initial set of guessed values, we adjust the parameters by hand until the model simulations agree with the experimental observations (Potapova et al., 2011). Once they agree, we stop parameter twiddling. In this regard, our current parameter set is a “working set” but by no means optimal, we supply the model code for people who are interested in further optimizing the parameters.

Time series simulations and bifurcation analysis of the model are carried out with XPPAUT, a freely available software (http://www.math.pitt.edu/~bard/xpp/xpp.html).

For the model simulations, we assume that nuclear envelope breaks when Cdk1:CycB has phosphorylated the majority (≥70%) of the mitotic substrates. We reduce ε to 25% at the time of NEBD.

We assume that the triggering signal of mitotic entry is the accumulation of CycB. Figure 5B of the experimental paper (Potapova et al., 2011) indicates that CycB is mainly produced during G2 phase. Furthermore, CycB production seems to be blocked after the cell enters mitosis or when the Wee1/Myt1 inhibitor is added. We set the model simulations accordingly. CycB level increases from 0.5 to one in absence of Wee1/Myt1 inhibitor, and CycB level is kept at 0.5 in the presence of Wee1/Myt1 inhibitor.

The experimental paper has reported measurements of H1 kinase activity and of pre-MPF. In our model, we represent H1 kinase activity by the ratio between the active, unphosphorylated Cdk1:CycB and the total CycB. Pre-MPF is represented by the ratio between phosphorylated Cdk1:CycB and the total CycB.

## Supplementary Material

Supplementary Material

## References

[b1] Arooz T. Yam C. H. Siu W. Y. Lau A. Li K. K. Poon R. Y. (2000). On the concentrations of cyclins and cyclin-dependent kinases in extracts of cultured human cells. Biochemistry 39, 9494–9501 10.1021/bi0009643 10924145

[b2] Blake-Hodek K. A. Williams B. C. Zhao Y. Castilho P. V. Chen W. Mao Y. Yamamoto T. M. Goldberg M. L. (2012). Determinants for activation of the atypical AGC kinase Greatwall during M phase entry. Mol. Cell. Biol. 32, 1337–1353 10.1128/MCB.06525-11 22354989 PMC3318580

[b3] Booher R. N. Holman P. S. Fattaey A. (1997). Human Myt1 is a cell cycle-regulated kinase that inhibits Cdc2 but not Cdk2 activity. J. Biol. Chem. 272, 22300–22306 10.1074/jbc.272.35.22300 9268380

[b4] Brizuela L. Draetta G. Beach D. (1989). Activation of human CDC2 protein as a histone H1 kinase is associated with complex formation with the p62 subunit. Proc. Natl. Acad. Sci. USA 86, 4362–4366 10.1073/pnas.86.12.4362 2543971 PMC287269

[b5] Burgess A. Vigneron S. Brioudes E. Labbé J.-C. Lorca T. Castro A. (2010). Loss of human Greatwall results in G2 arrest and multiple mitotic defects due to deregulation of the cyclin B-Cdc2/PP2A balance. Proc. Natl. Acad. Sci. USA 107, 12564–12569 10.1073/pnas.0914191107 20538976 PMC2906566

[b6] Charvin G. Oikonomou C. Siggia E. D. Cross F. R. (2010). Origin of irreversibility of cell cycle start in budding yeast. PLoS Biol. 8, e1000284 10.1371/journal.pbio.1000284 20087409 PMC2797597

[b7] Clarke P. R. Hoffmann I. Draetta G. Karsenti E. (1993). Dephosphorylation of cdc25-C by a type-2A protein phosphatase: specific regulation during the cell cycle in Xenopus egg extracts. Mol. Biol. Cell 4, 397–411. 8389619 10.1091/mbc.4.4.397 PMC300941

[b8] Cross F. R. Archambault V. Miller M. Klovstad M. (2002). Testing a mathematical model of the yeast cell cycle. Mol. Biol. Cell 13, 52–70 10.1091/mbc.01-05-0265 11809822 PMC65072

[b9] Dalal S. N. Schweitzer C. M. Gan J. DeCaprio J. A. (1999). Cytoplasmic localization of human cdc25C during interphase requires an intact 14-3-3 binding site. Mol. Cell. Biol. 19, 4465–4479. 10330186 10.1128/mcb.19.6.4465 PMC104405

[b10] Domingo-Sananes M. R. Kapuy O. Hunt T. Novak B. (2011). Switches and latches: a biochemical tug-of-war between the kinases and phosphatases that control mitosis. Philos. Trans. R. Soc. B 366, 3584–3594 10.1098/rstb.2011.0087 PMC3203464 22084385

[b11] Draetta G. Piwnica-Worms H. Morrison D. Druker B. Roberts T. Beach D. (1988). Human cdc2 protein kinase is a major cell-cycle regulated tyrosine kinase substrate. Nature 336, 738–744 10.1038/336738a0 2462672

[b12] Drummond-Barbosa D. Spradling A. C. (2004). α-endosulfine, a potential regulator of insulin secretion, is required for adult tissue growth control in Drosophila. Dev. Biol. 266, 310–321 10.1016/j.ydbio.2003.10.028 14738879

[b13] Galaktionov K. Beach D. (1991). Specific activation of cdc25 tyrosine phosphatases by B-type cyclins: evidence for multiple roles of mitotic cyclins. Cell 67, 1181–1194 10.1016/0092-8674(91)90294-9 1836978

[b14] Gavet O. Pines J. (2010). Activation of cyclin B1-Cdk1 synchronizes events in the nucleus and the cytoplasm at mitosis. J. Cell Biol. 189, 247–259 10.1083/jcb.200909144 20404109 PMC2856909

[b15] Gharbi-Ayachi A. Labbé J.-C. Burgess A. Vigneron S. Strub J.-M. Brioudes E. Van-Dorsselaer A. Castro A. Lorca T. (2010). The substrate of Greatwall kinase, Arpp19, controls mitosis by inhibiting protein phosphatase 2A. Science 330, 1673–1677 10.1126/science.1197048 21164014

[b16] Hara M. Abe Y. Tanaka T. Yamamoto T. Okumura E. Kishimoto T. (2012). Greatwall kinase and cyclin B-Cdk1 are both critical constituents of M-phase-promoting factor. Nat. Commun. 3, 1059 10.1038/ncomms2062 22968705 PMC3658099

[b17] He E. Kapuy O. Oliveira R. A. Uhlmann F. Tyson J. J. Novák B. (2011). System-level feedbacks make the anaphase switch irreversible. Proc. Natl. Acad. Sci. USA 108, 10016–10021 10.1073/pnas.1102106108 21617094 PMC3116408

[b18] Heald R. McLoughlin M. McKeon F. (1993). Human wee1 maintains mitotic timing by protecting the nucleus from cytoplasmically activated Cdc2 kinase. Cell 74, 463–474 10.1016/0092-8674(93)80048-J 8348613

[b19] Hoffmann I. Clarke P. R. Marcote M. J. Karsenti E. Draetta G. (1993). Phosphorylation and activation of human cdc25-C by cdc2–cyclin B and its involvement in the self-amplification of MPF at mitosis. EMBO J. 12, 53–63. 8428594 10.1002/j.1460-2075.1993.tb05631.x PMC413175

[b20] Holt L. J. Krutchinsky A. N. Morgan D. O. (2008). Positive feedback sharpens the anaphase switch. Nature 454, 353–357 10.1038/nature07050 18552837 PMC2636747

[b21] Huber M. D. Gerace L. (2007). The size-wise nucleus: nuclear volume control in eukaryotes. J. Cell Biol. 179, 583–584 10.1083/jcb.200710156 17998404 PMC2080922

[b22] Joerger A. C. Fersht A. R. (2007). Structure-function-rescue: the diverse nature of common p53 cancer mutants. Oncogene 26, 2226–2242 10.1038/sj.onc.1210291 17401432

[b23] Källström H. Lindqvist A. Pospisil V. Lundgren A. Rosenthal C. K. (2005). Cdc25A localisation and shuttling: characterisation of sequences mediating nuclear export and import. Exp. Cell Res. 303, 89–100 10.1016/j.yexcr.2004.09.012 15572030

[b24] Kim M.-Y. Bucciarelli E. Morton D. G. Williams B. C. Blake-Hodek K. Pellacani C. Von Stetina J. R. Hu X. Somma M. P. Drummond-Barbosa D. (2012). Bypassing the Greatwall-Endosulfine pathway: plasticity of a pivotal cell-cycle regulatory module in Drosophila melanogaster and Caenorhabditis elegans. Genetics 191, 1181–1197 10.1534/genetics.112.140574 22649080 PMC3416000

[b25] Krek W. Nigg E. A. (1991). Mutations of p34cdc2 phosphorylation sites induce premature mitotic events in HeLa cells: evidence for a double block to p34cdc2 kinase activation in vertebrates. EMBO J. 10, 3331–3341. 1655418 10.1002/j.1460-2075.1991.tb04897.x PMC453060

[b26] Liu F. Stanton J. J. Wu Z. Piwnica-Worms H. (1997). The human Myt1 kinase preferentially phosphorylates Cdc2 on threonine 14 and localizes to the endoplasmic reticulum and Golgi complex. Mol. Cell. Biol. 17, 571–583. 9001210 10.1128/mcb.17.2.571 PMC231782

[b27] López-Avilés S. Kapuy O. Novák B. Uhlmann F. (2009). Irreversibility of mitotic exit is the consequence of systems-level feedback. Nature 459, 592–595 10.1038/nature07984 19387440 PMC2817895

[b28] McGowan C. H. Russell P. (1993). Human Wee1 kinase inhibits cell division by phosphorylating p34cdc2 exclusively on Tyr15. EMBO J. 12, 75–85. 8428596 10.1002/j.1460-2075.1993.tb05633.x PMC413177

[b29] Mochida S. Ikeo S. Gannon J. Hunt T. (2009). Regulated activity of PP2A-B55 delta is crucial for controlling entry into and exit from mitosis in Xenopus egg extracts. EMBO J. 28, 2777–2785 10.1038/emboj.2009.238 19696736 PMC2750019

[b30] Mochida S. Maslen S. L. Skehel M. Hunt T. (2010). Greatwall phosphorylates an inhibitor of protein phosphatase 2A that is essential for mitosis. Science 330, 1670–1673 10.1126/science.1195689 21164013

[b31] Morgan D. (2006). The Cell Cycle: Principles Of Control London: New Science Press.

[b32] Novak B. Tyson J. J. (1993). Numerical analysis of a comprehensive model of M-phase control in Xenopus oocyte extracts and intact embryos. J. Cell Sci. 106, 1153–1168. 8126097 10.1242/jcs.106.4.1153

[b33] Novak B. Tyson J. J. Gyorffy B. Csikasz-Nagy A. (2007). Irreversible cell-cycle transitions are due to systems-level feedback. Nat. Cell Biol. 9, 724–728 10.1038/ncb0707-724 17603504

[b34] Parker L. L. Sylvestre P. J. Byrnes M. J. 3rd Liu F. Piwnica-Worms H. (1995). Identification of a 95-kDa WEE1-like tyrosine kinase in HeLa cells. Proc. Natl. Acad. Sci. USA 92, 9638–9642 10.1073/pnas.92.21.9638 7568188 PMC40857

[b35] Pomerening J. R. Sontag E. D. Ferrell J. E. Jr (2003). Building a cell cycle oscillator: hysteresis and bistability in the activation of Cdc2. Nat. Cell Biol. 5, 346–351 10.1038/ncb954 12629549

[b36] Pomerening J. R. Ubersax J. A. Ferrell J. E. Jr (2008). Rapid cycling and precocious termination of G1 phase in cells expressing CDK1AF. Mol. Biol. Cell 19, 3426–3441 10.1091/mbc.E08-02-0172 18480403 PMC2488275

[b37] Potapova T. A. Sivakumar S. Flynn J. N. Li R. Gorbsky G. J. (2011). Mitotic progression becomes irreversible in prometaphase and collapses when Wee1 and Cdc25 are inhibited. Mol. Biol. Cell 22, 1191–1206 10.1091/mbc.E10-07-0599 21325631 PMC3078080

[b38] Santos S. D. M. Wollman R. Meyer T. Ferrell J. E. Jr (2012). Spatial positive feedback at the onset of mitosis. Cell 149, 1500–1513 10.1016/j.cell.2012.05.028 22726437 PMC3395376

[b39] Sha W. Moore J. Chen K. Lassaletta A. D. Yi C.-S. Tyson J. J. Sible J. C. (2003). Hysteresis drives cell-cycle transitions in Xenopus laevis egg extracts. Proc. Natl. Acad. Sci. USA 100, 975–980 10.1073/pnas.0235349100 12509509 PMC298711

[b40] Sveiczer A. Novak B. Mitchison J. M. (1996). The size control of fission yeast revisited. J. Cell Sci. 109, 2947–2957. 9013342 10.1242/jcs.109.12.2947

[b41] Sveiczer A. Novak B. Mitchison J. M. (1999). Mitotic control in the absence of cdc25 mitotic inducer in fission yeast. J. Cell Sci. 112, 1085–1092. 10198290 10.1242/jcs.112.7.1085

[b42] Verdugo A. Vinod P. K. Tyson J. J. Novak B. (2013). Molecular mechanisms creating bistable switches at cell cycle transitions. Open Biology 3, 120179 10.1098/rsob.120179 23486222 PMC3718337

[b43] Voets E. Wolthuis R. M. F. (2010). MASTL is the human orthologue of Greatwall kinase that facilitates mitotic entry, anaphase and cytokinesis. Cell Cycle 9, 3591–3601 10.4161/cc.9.17.12832 20818157

[b44] Watanabe N. Broome M. Hunter T. (1995). Regulation of the human WEE1Hu CDK tyrosine 15-kinase during the cell cycle. EMBO J. 14, 1878–1891. 7743995 10.1002/j.1460-2075.1995.tb07180.x PMC398287

[b45] Yu J. Zhao Y. Li Z. Galas S. Goldberg M. L. (2006). Greatwall kinase participates in the Cdc2 autoregulatory loop in Xenopus egg extracts. Mol. Cell 22, 83–91 10.1016/j.molcel.2006.02.022 16600872

