# Supplementary material for: Robust mitotic entry is ensured by a latching switch
Source: Biol Open. 2013 Jul 26;2(9):924–31. doi: 10.1242/bio.20135199 (PMC3773339; doi:10.1242/bio.20135199)
Supplement: Supplementary Material [file supp_2_9_924__index.html]

Robust mitotic entry is ensured by a latching switch — Robust mitotic entry is ensured by a latching switch — Supplementary Material 

# Robust mitotic entry is ensured by a latching switch

## bio.20135199 Supplementary Material

**Files in this Data Supplement:**

- Supplementary Material - Chloe Tuck et al. doi: 10.1242/bio.20135199
