# Supplementary material for: Robust mitotic entry is ensured by a latching switch
Source: Biol Open. 2013 Jul 26;2(9):924–31. doi: 10.1242/bio.20135199 (PMC3773339; doi:10.1242/bio.20135199)
Supplement: Supplementary Material [file supp_bio.20135199_bio.20135199-s1.pdf]

Supplementary Material

Chloe Tuck et al. doi: 10.1242/bio.20135199

Biology Open

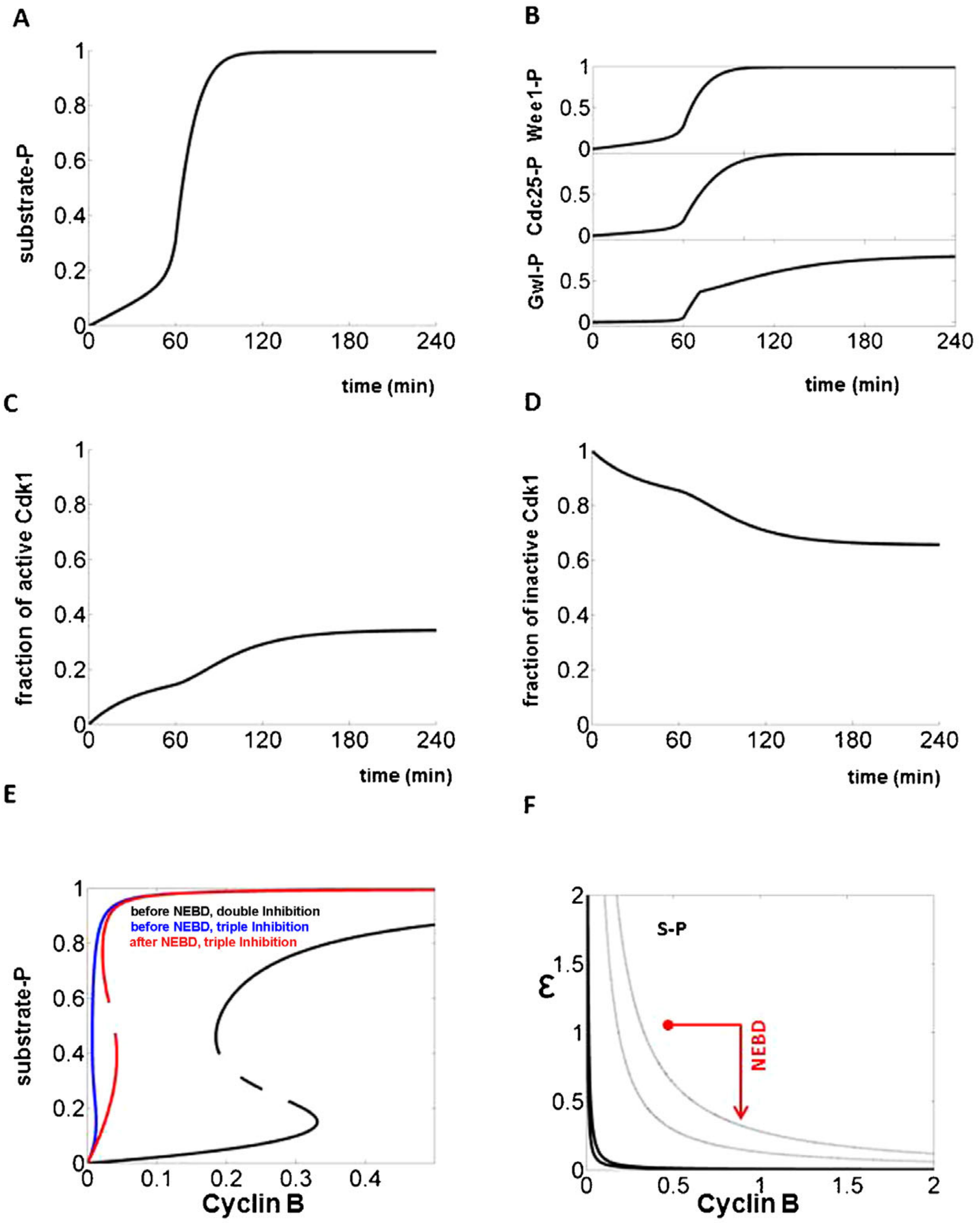

Fig. S1. Phosphatase inhibitor prevents mitotic collapse. Both Wee1/Myt1 and Cdc25 were applied at time zero, while phosphatase inhibitions only one hour later. Different panels are used to present the dynamics of different cell cycle regulators. The dynamics of mitotic substrate phosphorylation and pre-MPF (**A**,**D**) are directly comparable to experimental figure 6C of Potapova et al. (Potapova et al., 2011). The dynamics of phosphorylated Wee1, phosphorylated Cdc25, phosphorylated Gwl and H1 kinase activity (**B**,**C**) are model predictions. One (**E**) and two (**F**) parameter bifurcation diagrams. The boundaries of bistable region are shown before (Wee1/Myt1 and Cdc25 inhibition only, grey curves) and after phosphatase inhibition (black curves).

Table S1. XPPAUT ODE file for time series simulation and bifurcation analysis.

```
# To account for inhibition of Wee1/Myt1, Cdc25 and PP2A:B55, # ioa=iw=i25=0 in absence of inhibitor and ioa=iw=i25=25 in the presence of inhibitor.
b55a= b55/(1+ioa)
wee1a= wee1/(1+iw)
cdc25a= cdc25p/(1+i25)
# phosphorylation of MPF
k25 = kca25p*cdc25a + kca25*(cdc25t-cdc25a)
kwee = kciw*wee1a + kciwp*(wee1t-wee1a)
cycb' = k25*(cycbt-cycb) - kwee*cycb
wee1' = (kwa+kwa'*b55a)*(wee1t-wee1) - (kwi+kwi'*cycb)*wee1
cdc25p' = (k25a+k25a'*cycb)*(cdc25t-cdc25p) - (k25i+k25i'*b55a)*cdc25p
init wee1=1, cdc25p=0, cycb=0
# To mimic a G2 stage cell
# CyclinB production
cycbt' = ks
global 1 {cycbt-stop} {ks=0}
init cycbt=0.5, ks=0.0025
ks'=0
# For Bifurcation computation, set par cycbt=1 and comment out the cycbt ODE.
# [stop] is the level where CycB is sufficient for mitotic entry
# effect of nuclear envelope breakdown
sp' = (kp+kp'*cycb)*(st-sp)-(kdp+kdp'*b55a)*sp
gwp' = (kga+kga'*cycb*epsilon)*(gwt-gwp) - (kgi+kgi'*b55a)*gwp
epsilon'= 0
init epsilon=1
global 1 {sp-nebd} {epsilon=0.25}
init gwp=0
ensapt' = (kea+kea'*gwp)*(ensat-ensapt) - kdep*ensapt
init ensapt=0
b55' = (b55t-b55)*(kdep+kdis) - kbi*b55*(ensapt-b55t+b55)
init b55=1
# Parameter values
#total protein levels
p gwt=1, b55t=1, ensat=2, wee1t=1, cdc25t=1, st=1
#inhibitors
p ioa=0, iw=0, i25=0
# Rate constants
p kca25=0.005, kca25p=0.15, kciw=0.2, kciwp=0.02, kgi=0, kgi'=5, kga=0, kga'=0.5
p kea'=10, kea=0, kdep=0.3, kbi=50, kdis=1, kwa=0, kwa'=1, kwi=0, kwi'=1
p k25i=0, k25i'=1, k25a=0, k25a'=0.6, kp=0, kp'=1, kdp=0, kdp'=0.8
# Thresholds
p nebd=0.7, stop=1
# Numerical settings
@ yp=sp,xp=t,xlo=0,xhi=360,ylo=0,yhi=1
@ method=stiff,total=360,nplot=3,yp1=sp,yp2=gwl,yp3=cycb,dt=0.1
done
```
